# Multi-omics studies identified the molecular network of mitochondrial dysfunction in the pathogenesis of breast cancer

**DOI:** 10.1016/j.gendis.2026.102243

**Published:** 2026-05-17

**Authors:** Jinsong Liu, Xiao Guan, Liuliu Quan, Min Dou, Peng Yuan

**Affiliations:** a Department of Medical Oncology, National Cancer Center/National Clinical Research Center for Cancer/Cancer Hospital, Chinese Academy of Medical Sciences and Peking Union Medical College, Beijing 100021, China; b Department of VIP Medical Services, National Cancer Center/National Clinical Research Center for Cancer/Cancer Hospital, Chinese Academy of Medical Sciences and Peking Union Medical College, Beijing 100021, China; c State Key Lab of Molecular Oncology and Department of Pancreatic and Gastric Surgery, National Cancer Center/National Clinical Research Center for Cancer/Cancer Hospital, Chinese Academy of Medical Sciences and Peking Union Medical College, Beijing 100021, China; d Renhe Hospital, Shanghai University, Shanghai 201900, China

Mitochondrial dysfunction is implicated in breast cancer (BC) development, though its pathophysiological mechanisms remain incompletely understood.^1^ By integrating multi-omics data with Mendelian randomization and colocalization analysis, we identified significant associations between BC and i) DNA methylation at CpG sites in BCL2L11 (cg27608154) and MTERF1 (cg17054360), or ii) altered expression of ubiquinol-cytochrome C reductase core protein 1 (UQCRC1), mitochondrial pyruvate carrier 2 (MPC2), and mitochondrial ribosomal protein S18C (MRPS18C). Functional assays coupled with pathway analysis indicated that UQCRC1 suppressed BC proliferation and invasion by attenuating phosphatidylinositol-3-kinase (PI3K)/protein kinase B (AKT) signaling, identifying UQCRC1 as a promising potential therapeutic target for BC.

The study design and workflow were summarized in Figure. 1 Summary data for methylation, gene expression, and protein quantitative trait loci were obtained from public cohorts. Summary statistics of BC were sourced from the IEU GWAS project. Table S1 presents the causal associations between mitochondrial gene methylation and BC. After filtering (*P*-HEIDI > 0.01; *P*-SMR-FDR < 0.05), 33 CpG sites near 16 unique genes met marginal significance thresholds. Strong colocalization evidence (PPH4 > 0.70) implicated four genes in BC risk: BCL2L11 (cg27608154), BIK (cg01341487, cg04042468, cg26453588), MTERF1 (cg17054360), and TRMT1 (cg12014333, cg22237401) (Table S4; Fig. S1 and S2).

**Figure 1:**
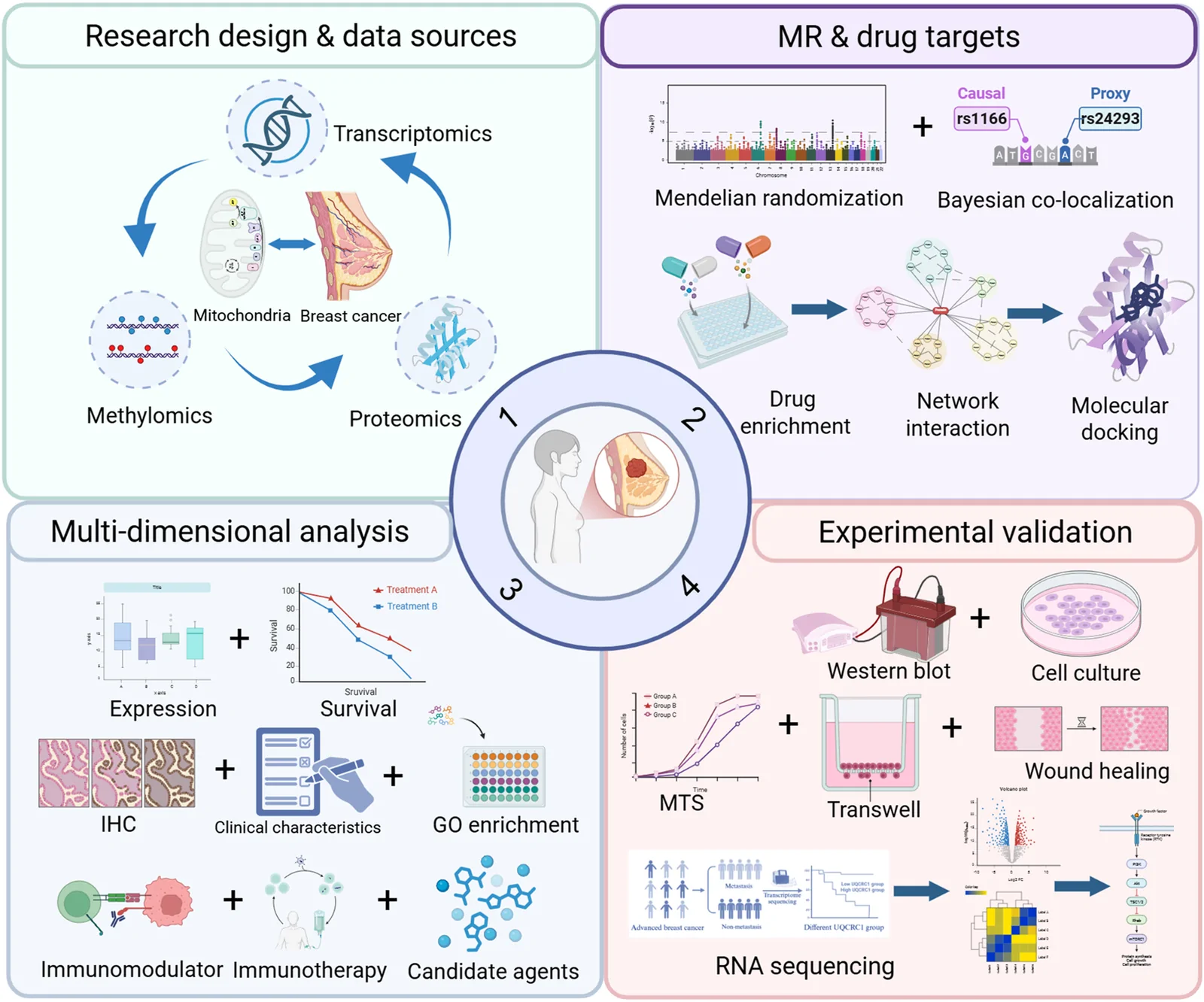
The design and workflow diagram of this study.

Causal associations for gene expression were detailed in Table S2. Summary-based Mendelian randomization (SMR) analysis with multiple testing correction (FDR-*P* < 0.05) identified 13 genes whose genetically predicted expression levels associated with BC risk: MTX1, MRPS18C, FDPS, HSCB, MPC2, MUTYH, UQCRC1, SURF1, MRPS16, NSUN2, NIT2, NUDT13, and COX5A.^2^ Colocalization analysis revealed robust evidence (PPH4 > 0.70) for three genes: UQCRC1, MPC2, and MRPS18C (Table S5 and Fig. S3). Table S3 summarizes causal associations for mitochondrial proteins. Nine proteins (KYAT3, FH, NIT2, PPA2, DNAJC30, NUDT2, ACADVL, DCXR, SARS2) met significance thresholds (*P*-HEIDI > 0.01; *P*-SMR < 0.05). No mitochondria-associated proteins retained a significant association with BC risk after multiple testing correction.

While Mendelian randomization/colocalization analyses identified no significant mitochondrial protein targets, three transcriptome-level genes (UQCRC1, MPC2, MRPS18C) emerged as potential therapeutic targets. Drug enrichment analysis (DSigDB) and protein–drug network construction (Cytoscape) identified: UQCRC1-targeting compounds: 1-Methyl-4-phenyl-2,3-dihydropyridinium, cube root extract, 635-65-4; MRPS18C-targeting compound: 4-phenylbutyric acid (Fig. S4A and S4B). Molecular docking confirmed ligand–receptor interactions with favorable binding energies (Table S6 and Fig. S4C–S4F), where lower energies indicate higher binding affinity.

Previous analyses suggested that UQCRC1 might be a potential drug target in BC. We first analyzed the expression level and clinicopathological characteristics of UQCRC1. The results showed that UQCRC1 had lower expression levels in BC with worse prognostic value, which was also confirmed by immunohistochemistry (Fig. S5A–S5C). The expression level of UQCRC1 in different BC subtypes was different, and with the increase of its expression, the stage and grade of the patients also decreased (Fig. S5D and S5E). This suggests that UQCRC1 might act as a protective factor. Finally, functional analysis also showed that UQCRC1 mainly played a crucial role in energy metabolism, RNA regulation, and mitochondrial activity (Fig. S5F). To further explore the biological role of UQCRC1 in BC, we first conducted immune infiltration and immune checkpoint inhibitor analysis, and the results showed that the expression of UQCRC1 was related to the immune infiltration of CD4^+^ T lymphocytes, mast cells, CD8^+^ T lymphocytes, and neutrophils (Fig. S6A and S6B). Moreover, immune checkpoint inhibitors, including CTLA4, CD276, and LAG3,^3^ were closely related to the expression of UQCRC1 (Fig. S6C and S6D). Clinical models of UQCRC1 have achieved good immunotherapy prediction efficacy in multiple cohorts (Fig. S6E). Correlation analysis between targeted therapy drugs and UQCRC1 showed that capivasertib and UQCRC1 might have a coordinated relationship in multiple cohorts, which could help solve the problem of capivasertib resistance (Fig. S6F and S6G). In addition, PI3KCA mutations were closely related to UQCRC1, which further validated the coordinated effect of PI3KCA inhibitors and UQCRC1 (Fig. S6H).

To assess the functional impact of identified targets on BC, we conducted cell proliferation and migration assays. After transfection with the Si-UQCRC1 plasmid, the protein level of UQCRC1 was significantly knocked down (Fig. S7A). In MCF7 and T47D BC cell lines, UQCRC1 knockdown significantly enhanced cell proliferation (MTS assay), migration (wound healing assay), and invasion (Transwell assay) compared with control groups (Fig. S7B–S7D). These results collectively demonstrated that UQCRC1 suppressed proliferation and migration in BC cells.

To elucidate the mechanism underlying UQCRC1's role in BC progression, RNA sequencing was performed on metastatic versus non-metastatic BC patient tissues (Fig. S8A). Patients were stratified into high and low UQCRC1 groups based on median expression. Differential expression analysis between these groups, followed by enrichment analysis of differentially expressed genes, revealed significant enrichment in metabolic pathways, PI3K/AKT, and mTOR signaling (Fig. S8B–S8D). Western blotting analysis further demonstrated elevated phosphorylated PI3K (P-PI3K) and AKT (P-AKT) levels in UQCRC1-knockdown cells, while total PI3K and AKT protein abundance remained unchanged (Fig. S8E). These findings indicated that UQCRC1 inhibited BC proliferation and migration by suppressing activation of the PI3K/AKT signaling pathway.

In conclusion, this study suggested that mitochondria-associated gene regulation influenced breast cancer susceptibility at methylation, gene expression and protein levels. Meanwhile, the integration of multi-omics data has also identified the potential of UQCRC1 as a biomarker and therapeutic target for breast cancer.

## CRediT authorship contribution statement

**Jinsong Liu:** Writing – original draft, Formal analysis. **Xiao Guan:** Formal analysis, Data curation. **Liuliu Quan:** Data curation. **Min Dou:** Writing – review & editing. **Peng Yuan:** Writing – review & editing.

## Ethics declaration

The study was approved by the Ethics Review Committee, Cancer Hospital of the Chinese Academy of Medical Sciences (Approval No. 22/534–3736), and informed consent was obtained from the patient.

## Data availability

The original information supporting the assay's conclusions can be obtained by getting in touch with the author.

## Funding

This work was supported by the 10.13039/501100001809National Natural Science Foundation of China (No. 82172650) and the Capital Health Development and Scientific Research Special Project (China) (No. 2024-1-4021).

## Conflict of interests

The authors state that the research has no conflict of interests.
