# Supplementary material for: Multi-omics studies identified the molecular network of mitochondrial dysfunction in the pathogenesis of breast cancer
Source: Genes Dis. 2026 May 17;14(2):102243. doi: 10.1016/j.gendis.2026.102243 (PMC13634571; doi:10.1016/j.gendis.2026.102243)
Supplement: Supplementary file 1 [file mmc1.docx]

1. **Introduction**

Breast cancer represents a major global health burden, ranking second in incidence and fourth in mortality among all cancers [1]. Despite available treatments including surgery, chemotherapy, targeted therapy, and novel antibody-drug conjugates (ADCs), a significant proportion of patients present with advanced disease or experience recurrence and metastasis [2, 3]. Early detection is crucial, yet current tumor markers suffer from limited specificity, underscoring the critical need for novel diagnostic targets.

Mitochondria provide most of the energy needed for cellular metabolism. Alterations in mitochondrial function are one of the critical markers of tumors, which usually manifest as abnormal mitochondrial morphology, insufficient mitochondrial copy number, abnormal energy metabolism and mitophagy [4]. Mitochondrial maintain energy metabolism homeostasis, regulate ATP production, ROS production, and carbon flow allocation, and profoundly affect the proliferation and metastasis of breast cancer. Breast cancer cells exhibit amazing metabolic plasticity, with MCF-7 cells typically dependent on oxidative phosphorylation (OXPHOS), while MDA-MB-231 cells exhibit a mixed metabolic state in which glycolysis coexists with OXPHOS. This plasticity allows cancer cells to survive in a nutrient-deprived tumor microenvironment and provides energy security for the metastatic process. Exploring the key functions played by mitochondria in breast cancer and delving into the underlying mechanisms of their action paves the way for the identification of therapeutic targets for breast cancer. Beyond the 37 core bioenergetic genes encoded within mitochondrial DNA (mtDNA), the nuclear genome harbors over 1,000 additional genes involved in mitochondrial function. Consequently, numerous experimental and epidemiological studies have focused on mtDNA to investigate potential causal links between mitochondrial dysfunction and cancer. However, the results produced by these studies were inconsistent and did not take into account the effects of confounding factors to distinguish between cause and effect. And there is no in-depth verification in combination with experiments. Therefore, a strong strategy and integrated assay for thorough study of all genes linked to mitochondrial dysfunction are required.

Mendelian randomization is a technique that investigates possible causal links between exposure and results by using genetic variation as an instrumental variable. This method could reverse causality bias. The idea of MR has been broadened and developed by SMR, which uses QTL data and independent GWAS pooled statistics to rank possibly harmful genes among GWAS findings. Heterogeneity testing and colocalization analysis were followed to increase the reliability of the results. The study of molecular docking and drug targets could further explore the biological functions of targets in tumors.

This study integrated multi-omics data from large-scale GWAS and multidimensional QTL analyses to investigate mitochondrial gene associations with breast cancer using SMR and colocalization. Subsequent drug enrichment analysis identified potential interactions between novel therapeutic agents and mitochondrial targets. Clinicopathological characteristics and immune correlates of key targets were analyzed. Functional validation through *in vitro* assays, RNA sequencing of clinical specimens, and pathway enrichment analyses confirmed candidate therapeutic targets for breast cancer while providing a mechanistic foundation for further investigation.

2. **Materials and Methods**
   2.1 **Omics and cohort data sources**

To investigate the association between mitochondrial gene regulation and BC susceptibility, we integrated multi-omics data (**Figure 1**). Mitochondrial genes were sourced from MitoCarta 3.0. Publicly available datasets encompassing DNA methylation, gene expression, and protein abundance were analyzed to comprehensively assess mitochondrial gene involvement in BC. Quantitative trait loci (QTLs) elucidate associations between single nucleotide polymorphisms (SNPs) and methylation (mQTLs), expression (eQTLs), or protein levels (pQTLs). Blood mQTLs: McRae et al. provided SNP-CpG associations derived from mQTL analysis of 1980 individuals of European ancestry. Methylation probe normalization employed a generalized linear model, incorporating chip correction. Blood eQTLs: The eQTLGen consortium dataset (31,684 individuals) served as the source for blood expression QTLs. pQTLs: Summary statistics for circulating protein levels were obtained from Ferkingstad et al.'s study involving 35,559 Icelandic cases. BC susceptibility GWAS: BC risk summary statistics originated from the ieu-a-1127 dataset (Combined Oncoarray; iCOGS; 69,501 cases, 105,974 controls).

2.2 **Summary-data-based MR analysis**

We employed SMR to estimate causal effects of mitochondrial gene methylation, expression, and protein on BC risk. Leveraging the strongest cis-QTLs as instrumental variables, SMR enhances statistical power over conventional MR when GWAS data derive from large samples. For each gene, cis-QTLs were defined within a±1000 kb window, selecting variants surpassing *P*-value threshold of 5.0×10^-8^. The Heterogeneity in Dependent Instruments (HEIDI) test distinguished linkage from pleiotropy. Analyses utilized SMR software with default parameters. *P*-values underwent Benjamini-Hochberg correction to control the false discovery rate (FDR) below 0.05. Colocalization required FDR *P*-values < 0.05 and *P*-HEIDI > 0.01.

2.3 **Colocalization analysis**

Causal variant sharing between mitochondrial mQTLs, eQTLs, or pQTLs and BC was assessed using the coloc R package. This method calculates posterior probabilities for five hypotheses: H0: No causal variant for either trait. H1: Causal variant for the molecular trait (expression/methylation/protein) only. H2: Causal variant for BC only. H3: Distinct causal variants for each trait. H4: Shared causal variant for both traits. Region windows for colocalization were set to ±1000 kb (pQTL/eQTL-GWAS) and ±500 kb (mQTL-GWAS), consistent with established protocols. A posterior probability for H4 (PPH4)> 0.70 provided evidence supporting colocalization and strengthening causal inference.

2.4 **Druggability analysis**

Drug target enrichment analysis utilized DSigDB, a gene set resource linking drugs/compounds was used for Gene Set Enrichment Analysis. This database contains 22,527 gene sets representing 17,389 compounds targeting 19,531 genes. We downloaded the "DSigDBv1.0 Detailed.txt" file and performed enrichment analysis.

2.5 **Protein-protein interaction (PPI)**

The STRING database (https://cn.string-db.org/) was employed to construct and analyze PPI networks for identified drug targets.

2.6 **Molecular docking**

For drugs with confirmed targets, small molecule structures were retrieved from PubChem. Corresponding protein 3D structures were sourced from the Protein Data Bank and the AlphaFold Protein Structure Database. Molecular docking was performed using CB-Dock2, a method that automatically identifies binding sites, calculates docking box parameters based on the ligand, and employs AutoDock Vina. Binding energy, reflecting receptor-ligand affinity, was calculated; values < -5 kcal/mol indicate potential binding.

2.7 **Expression and clinicopathological characteristics analysis**

The UALCAN database was used to explore the protein expression level of UQCRC1 in pan-cancer species and survival analysis was conducted with TIMER2. In order to further verify the expression level of UQCRC1 in breast cancer, the Human Protein Atlas was performed. Subsequently, clinicopathological features analysis included typological expression, staging and hierarchical was conducted.

2.8 **Immune infiltration and therapeutic analysis**

Based on a variety of immune algorithms including EPIC and cibersort, UQCRC1 and immune cells, immunoassay inhibitors were explored with R packet “cibersort, GfellerLab/EPIC”.

2.9 **Clinical mutation and drug candidate screening**

Clinical mutations and targeted drugs were identified using the BEST database (<https://rookieutopia.hiplot.com.cn/app_direct/BEST/>).

2.10 **Cell culture and and tissue samples**

Breast cancer cell lines were supplied by the American Type Culture Collection (ATCC, USA). The cells were cultured in RPMI-1640 or RPMI-DMEM (Gibco) containing 10% FBS with 5% CO2 at 37°C. Cancer Hospital, Chinese Academy of Medical Sciences (CHCAMS) provided breast cancer tissue samples. Informed consent was acquired from patients. The local ethics commission gave its approval to this study.

2.11 **Plasmid transfection**

SiRNA, including NC, Si-UQCRC1-1 and Si-UQCRC1-2, were obtained from Suzhou GenePharma Co., Ltd. Transfection was performed with Lipofectamine 3000 (Invitrogen, USA). Briefly, cells were cultured in 6-well plates and when the cell density reached 70%, transfected with NC, Si-UQCRC1-1 and Si-UQCRC1-2 and cultured for 48h. The detailed transfection procedure has been described previously.(Si-1：CAUCUGGCUUUCAAGGGAATTUUCCCUUGAAAGCCAGAUGTT；

Si-2: GUGCACACUUUGUCUGUGATTUCACAGACAAAGUGUGCACTT).

2.12 **Western blot**

RIPA lysis buffer (Sangon Biotech) was used to extract proteins from breast cancer cells. We used 10% SDS-PAGE to separate proteins and transferred the proteins to PVDF membranes (Millipore). Then, the PVDF were incubated with a UQCRC1 antibody (Proteintech), P-PI3K antibody (Proteintech), P-AKT antibody (Proteintech), PI3K/AKT antibody (Proteintech) and β-actin antibody (Proteintech) at 4°C overnight and incubated with a goat IgG HRP antibody (Proteintech) for 2 h. An ECL detection kit (Labgic) was used to visualize the protein bands.

2.13 **MTS assay**

Cell proliferation was performed using MTS assay. After Si-UQCRC1-1 and Si-UQCRC1-2 transfection, breast cancer cells were seeded into 96-well plates and incubated for 0, 24, 48 and 72 h. the well contents were added with MTS reagent, incubated for 1 h. A microplate reader was used to measure the optical density at 490 nm.

2.14 **Wound healing assay**

A six-well plate was seeded with transfected breast cancer cells. when the cell density reached 90%, a wound was scratched. After incubation for 0, 24, and 48 h, the wounded areas were recorded by microscopy, and the percentage of wound healing area was calculated with Image J.

2.15 **Transwell assay**

Transfected cells were cultured in the upper chamber (Corning, USA) with serum-free medium, and complete medium containing 20% FBS in the lower chamber. After 24 h, the cells were fixed for 30 min and stained with for 0.5 h. The cells were counted after imaging by microscopy.

2.16 **RNA sequencing**

RNA-seq for the observed transcriptomic profile of 18 breast cancer tissue samples was performed using Illumina® (Wuhan Seqhealth Co., Ltd., China) according to instructions of the manufacturer. RNA sequencing was carried out as previously described.

2.17 **Differential expression analysis**

Limma is a differential expression screening method, and we perform differential analysis between different comparator groups and controls.

2.18 **Pathway enrichment analysis**

Functional enrichment analysis of gene sets was performed to retrieve the most current KEGG pathway gene annotations. Enrichment analysis was subsequently conducted employing the clusterProfiler R package to identify significantly enriched pathways.

2.19 **Statistical analysis**

GraphPad Prism 9.0 and R software (version 4.3.1) were used. ImageJ was performed to calculate the cell number and area. The mean ± standard deviation is shown for all statistical results. Multiple t test was used.

3. **Discussion**

Breast cancer is the most common female tumor, and the recurrence and metastasis rate are still high at 5-10 years. Meanwhile, mitochondria serve as the hub of metabolic reprogramming in breast cancer, driving tumor progression through multi-dimensional mechanisms such as kinetic abnormalities, bioenergetic remodeling, and microenvironment interactions. The strategy of targeting mitochondria-related pathways has opened up a new avenue for precision treatment of breast cancer. In this study, after multi-omics analyses, at the mQTL level, we found the methylation of cg27608154 in BCL2L11, the methylation of cg17054360 in MTERF1, and the methylation of cg01341487, cg04042468, cg26453588 in BIK were associated with breast cancer risk. At eQTL levels, UQCRC1, MPC2, and MRPS18C were strongly associated with breast cancer. However, genetic prediction without mitochondria-associated proteins is associated with breast cancer risk after correction for multiple testing.

There are three mitochondrial genes that have been validated at the transcriptome level by multi-omics to be strongly associated with breast cancer, and could serve as potential therapeutic targets. MPC2 (Mitochondrial Pyruvate Carrier 2) is located in the inner mitochondrial membrane. Diseases associated with MPC2 include mitochondrial pyruvate carrier defects and apical myocardial infarction. The relevant pathways include glycolysis and regulation of pyruvate metabolism. Gene ontology annotation associated with this gene includes pyruvate transmembrane transporter activity. Agata Sofia Assuncao Carreira et al. found that MPC2 was revealed to be a key regulator of triple-negative breast cancer cells adapting to NAD(H) shortage. When NAD(H) levels are insufficient, MPC2 expression is down-regulated, prompting cancer cells to reduce mitochondrial pyruvate uptake, which in turn enhances glycolysis and glutamine metabolism to maintain energy supply and support tumor growth. This metabolic reprogramming enhances the ability of cancer cells to survive redox stress. Peng-Cheng Zhong et al. found that the expression of mitochondrial pyruvate carriers MPC1 and MPC2 was the lowest in quiescent tumors, while MPC2 was higher in cholesterol-derived tumors than glycolytic or quiescent tumors. Of course, the mechanism of MPC2 in metabolic isoforms needs to be further explored.

MRPS18C (Mitochondrial Ribosomal Protein S18C) is a mitochondrial ribosomal protein-coding gene. Conditions associated with MRPS18C include dyskinesia, cerebral palsy, and Hermansky-Pudlak syndrome. Relevant pathways include mitochondrial translation and protein metabolism. Gene ontology annotation associated with this gene includes the structural composition of ribosomes. Xiaoyi Lin et al. found that MRPS18C was involved in mitochondrial translation termination and translation during the construction of a prognostic model of mitochondrial ribosomal protein, and its high expression indicated a poor prognosis in BC patients. However, the detailed regulatory model of MRPS18C in mitochondrial regulation needs to be further studied.

UQCRC1 (Ubiquinol-Cytochrome C Reductase Core Protein 1) is a protein-coding gene. Conditions associated with UQCRC1 include parkinsonism with polyneuropathy and metaphyseal dysplasia of the spine. The relevant pathways include respiratory electron transport and complex III assembly. In the process of breast cancer occurrence and development, the regulatory mode of UQCRC1 in mitochondrial regulation is still blank, and in-depth experimental exploration is needed.

Meanwhile, molecular docking results showed novel drugs including 1-Methyl-4-phenyl-2,3-dihydropyridinium, 635-65-4, and cube root extract could target UQCRC1 to function, while, 4-PHENYLBUTYRIC ACID could target MRPS18C to function. Importantly, these molecular docking findings are in silico predictions and should be interpreted as hypothesis-generating; thus, the listed compounds are candidate agents that require further experimental validation (e.g., binding/target engagement assays and functional studies) before any therapeutic claims can be made. J R Cashman found that 1-Methyl-4-phenyl-2,3-dihydropyridinium was produced during cellular metabolism and had an impact on the development of cancer, but its role in breast cancer was unknown. Moreover, the core active ingredient of Cube Root Extract is rotenone compounds. It exerts potential anti-tumor effects through the following mechanisms: inhibition of mitochondrial respiratory chain complex I, blocking ATP synthesis, leading to the collapse of cancer cell energy; Induce the burst of reactive oxygen species and trigger oxidative stress damage; Activates apoptotic pathways to selectively induce tumor cell death. Experiments have shown that it has significant inhibitory activity against a variety of cancer cell lines, but its selectivity and in vivo efficacy need to be further verified. In breast cancer research, 4-phenylbutyric acid (4-PBA) mainly exerts anti-tumor effects as an endoplasmic reticulum stress inhibitor and histone deacetylase (HDAC) inhibitor: Inhibition of endoplasmic reticulum stress: alleviate the pressure of tumor microenvironment and enhance chemotherapy sensitivity; Epigenetic regulation: restore the expression of tumor suppressor genes through HDAC inhibition, and inhibit the proliferation of triple-negative breast cancer; Regulate the immune microenvironment: reduce the secretion of pro-inflammatory factors and inhibit tumor metastasis. In vitro and animal models have confirmed that it could enhance the efficacy of traditional therapies, but clinical translation needs to be further validated. These are the theoretical references for the study of mitochondria in breast cancer.

Finally, we conducted experiments on mitochondria-related genes including UQCRC1, MPC2, and MRPS18C, and found that UQCRC1 played a critical role in the occurrence and development of breast cancer. Function experiments indicated UQCRC1 inhibited the proliferation and migration of breast cancer cells. RNA sequencing and functional pathway enrichment analysis suggested that UQCRC1 could inhibit the proliferation and migration of breast cancer by inhibiting the PI3K/AKT signaling pathway. Notably, as UQCRC1 is a core component of mitochondrial respiratory chain complex III, the link between mitochondrial dysfunction and cytoplasmic PI3K/AKT activation is biologically plausible and may be mediated by mitochondrial stress signals. Specifically, UQCRC1 depletion could increase mitochondrial ROS production, compromise oxidative phosphorylation and ATP availability, and/or perturb mitochondrial-associated metabolites (e.g., succinate or fumarate), which have been reported to act as signaling intermediates that modulate kinase cascades and cellular proliferation/migration programs. Therefore, we interpret the observed PI3K/AKT pathway changes as consistent with a mitochondria-to-cytosol signaling axis, and future work will directly quantify mitochondrial ROS (e.g., MitoSOX), ATP levels, mitochondrial membrane potential, and key metabolites to mechanistically connect UQCRC1/complex III dysfunction to PI3K/AKT/mTOR phosphorylation.

A key strength of this study lies in the complementary application of SMR and colocalization analyses. These methods leverage genetic variation to infer causal effects of mitochondrial gene regulation, effectively mitigating potential confounding from linkage disequilibrium. Furthermore, the integration of large-scale GWAS data significantly enhanced statistical power. The multi-omics evidence convergence robustly supports the causal association between mitochondria-associated genes and breast cancer susceptibility. Several limitations warrant acknowledgment. First, incomplete proteome coverage within the pQTL dataset constrained our ability to comprehensively assess causal relationships between mitochondrial proteins and breast cancer risk. Second, the functional role of UQCRC1 was not validated in vivo using animal models. Investigating UQCRC1's mechanism of action in vivo constitutes a primary focus for subsequent investigation. Third, although our in vitro assays support a tumor-suppressive role of UQCRC1, the upstream mitochondrial biology linking UQCRC1/complex III dysfunction to downstream signaling remains to be further grounded by direct measurements, which will be prioritized in follow-up studies. From a clinical perspective, the convergent genetic and multi-omics evidence nominates UQCRC1 (and other prioritized mitochondrial regulators) as candidate biomarkers for risk stratification and prognosis, and as potential therapeutic vulnerabilities; however, clinical translation will require validation in larger independent patient cohorts, standardized detection, and evaluation of treatment-response associations. Future work will therefore focus on (i) expanding proteomic coverage and incorporating additional QTL resources from broader clinical validation, (ii) validating UQCRC1 function and pathway modulation *in vivo* (e.g., xenograft or orthotopic models), and (iii) testing candidate compounds/targeting strategies with rigorous target-engagement and efficacy assessments before clinical consideration.

4. **Conclusions**

This study suggested that mitochondria-associated gene regulation influenced breast cancer susceptibility at methylation, gene expression and protein levels. Meanwhile, the integration of multi-omics data has also identified the potential of UQCRC1 as a biomarker and therapeutic target for breast cancer.

**Figure legends:**

**Figure S1** Colocalization analysis between CpG cg27608154 (BCL2L11)/cg01341487 (BIK)/cg04042468 (BIK)/ cg26453588 (BIK) mQTLs and breast cancer GWAS in the locuszoom plots. The y-axis shows –log10 (*P* value) of mQTL association tests. Points are color-coded based on LD (r2) relative to the highlighted variant.

**Figure S2** Colocalization analysis between CpG cg17054360 (MTERF1)/cg12014333 (TRMT1)/cg22237401 (TRMT1) mQTLs and breast cancer GWAS.

**Figure S3** Colocalization results between UQCRC1/MPC2/MRPS18C and breast cancer GWAS.

**Figure S4** **(A, B)** Drug enrichment and drug–protein network interactions in mitochondrial genes. **(C–F)** Molecular docking analysis between novel drugs and mitochondrial genes.

**Figure S5** **(A)** The expression level of UQCRC1 across cancers in CPTAC samples. **(B)** Survival analysis of UQCRC1 in TIMER2. **(C)** Protein expression of UQCRC1 in breast cancer and normal samples. **(D, E)** Clinicopathological characteristics of UQCRC1 in various datasets. **(F)** Go enrichment analysis of UQCRC1.

**Figure S6** **(A, B)** Immunoinfiltration analysis of UQCRC1 in multiple cohorts. **(C, D)** The association of UQCRC1 with immune checkpoints in multiple cohorts. **(E)** Clinical immune prediction of UQCRC1 in multiple cohorts. **(F, G)** Candidate agents screening of UQCRC1 in multiple cohorts. **(H)** Genomics alteration landscape of UQCRC1.

**Figure S7** UQCRC1 could inhibit the proliferation and migration of breast cancer cells. **(A)** Protein levels of UQCRC1 in UQCRC1 knockdown and control. **(B)** UQCRC1 significantly inhibited the proliferation of MCF7 and T47D cells in the MTS assay. **(C)** UQCRC1 significantly inhibited the migration of MCF7 and T47D cells in the wound healing assay. **(D)** UQCRC1 significantly inhibited the invasion of MCF7 and T47D cells in the Transwell assay. Scale bar: 50 μm. *n* = 3 independent experiments.

**Figure S8** UQCRC1 could inhibit the PI3K/AKT signaling pathway. **(A)** Transcriptome sequencing results in patients with metastatic and non-metastatic breast cancer. **(B, C)** Volcano and heat maps demonstrating differential genes in the high and low UQCRC1 groups. **(D)** Functional pathway enrichment analysis of the above differential genes. **(E)** Protein levels of UQCRC1, AKT, PI3K, P-AKT, and P-PI3K in UQCRC1 knockdown and control. Scale bar: 50 μm.

**Supplementary Table 1:** SMR analysis for association between mitochondrial gene methylations and breast cancer.

**Supplementary Table 2:** SMR analysis for association between mitochondrial gene expression and breast cancer.

**Supplementary Table 3:** SMR analysis for association between mitochondrial gene encoded protein and breast cancer.

**Supplementary Table 4:** Colocalization results for association between mitochondrial gene methylations and breast cancer.

**Supplementary Table 5:** Colocalization results for association between mitochondrial gene expression and breast cancer.

**Supplementary Table 6:** Drug enrich outcomes of mitochondrial gene.

**Reference:**

1.	Bray, F., et al., *Global cancer statistics 2022: GLOBOCAN estimates of incidence and mortality worldwide for 36 cancers in 185 countries.* CA Cancer J Clin, 2024. **74**(3): p. 229-263.

2.	Polyak, K., *Heterogeneity in breast cancer.* J Clin Invest, 2011. **121**(10): p. 3786-8.

3.	Galluzzi, L., et al., *Molecular mechanisms of cell death: recommendations of the Nomenclature Committee on Cell Death 2018.* Cell Death Differ, 2018. **25**(3): p. 486-541.

4.	Bao, X., et al., *The crosstalk between HIFs and mitochondrial dysfunctions in cancer development.* Cell Death Dis, 2021. **12**(2): p. 215.
